# Novel Compound Heterozygous Variants in *TBCD* Gene Associated with Infantile Neurodegenerative Encephalopathy

**DOI:** 10.3390/children8121140

**Published:** 2021-12-05

**Authors:** Chih-Ling Chen, Chien-Nan Lee, Yin-Hsiu Chien, Wuh-Liang Hwu, Tung-Ming Chang, Ni-Chung Lee

**Affiliations:** 1 Department of Medical Genetics, National Taiwan University Hospital, Taipei 100226, Taiwan; 111811@ntuh.gov.tw (C.-L.C.); leecn@ntu.edu.tw (C.-N.L.); chienyh@ntu.edu.tw (Y.-H.C.); hwuwlntu@ntu.edu.tw (W.-L.H.); 2 Department of Obstetrics and Gynecology, National Taiwan University Hospital, Taipei 100226, Taiwan; 3 Department of Pediatrics, National Taiwan University Hospital, Taipei 100226, Taiwan; 4 Department of Pediatrics, Changhua Christian Hospital, Changhua 50006, Taiwan; 5 Department of Biological Science and Technology, College of Biological Science and Technology, National Yang Ming Chiao Tung University, Hsinchu 300093, Taiwan

**Keywords:** *TBCD*, tubulinopathy, neurodegeneration, biallelic

## Abstract

Mutations in tubulin-specific chaperon D (TBCD), the gene encoding one of the co-chaperons required for the assembly and disassembly of the α/β-tubulin heterodimers, have been reported to cause perturbed microtubule dynamics, resulting in debilitating early-onset progressive neurodegenerative disorder. Here, we identified two novel *TBCD* variants, c.1340C>T (p.Ala447Val), and c.817+2T>C, presented as compound heterozygotes in two affected siblings born to unaffected carrier parents. Clinical features included early-onset neurodegeneration, failure to thrive, respiratory failure, hypotonia, muscle weakness and atrophy and seizures. We established the genotype–phenotype relationship of these *TBCD* pathogenic variants and provided insight into the protein structural alteration that may contribute to this chaperone-associated tubulinopathy.

## 1. Introduction

Microtubule dynamic plays a pivotal role in neuronal development and function. The organization of mitotic spindles involves the polymerization and depolymerization of αβ-tubulin heterodimers in the microtubule-organizing center (MTOC). Biallelic loss-of-function mutations in tubulin-specific chaperon D (TBCD), the gene encoding one of the co-chaperons involved in the assembly–disassembly processes, have been reported to cause perturbed microtubule dynamics [1,2]. The recessive inheritance of *TBCD* mutations may result in early-onset neurodegenerative encephalopathy, as reported in the previous literature (OMIM #604649). This rare disease is characterized by early-onset diffuse brain atrophy, postnatal microcephaly, failure to thrive, respiratory failure, hypotonia, muscle weakness and atrophy, seizures, spasticity, regression, developmental delay, profound intellectual disability, and absent visual tracking [3]. The majority of affected patients showed an early onset of symptoms before 1 year of age and had often required respiratory support since infancy. No affected individual living beyond the age of 20 has been reported to date [3]. Brain imaging studies of the affected cases showed evidence of hypomyelination, rudimentary corpus callosum, and progressive brain atrophy involving cerebrum, cerebellum, and brain stem [2,3,4]. Here, we report two novel *TBCD* variants presented as heterozygous compound mutations in two affected siblings born to non-consanguineous carrier parents of Chinese descent.

## 2. Case Presentation

**Figure 1:**
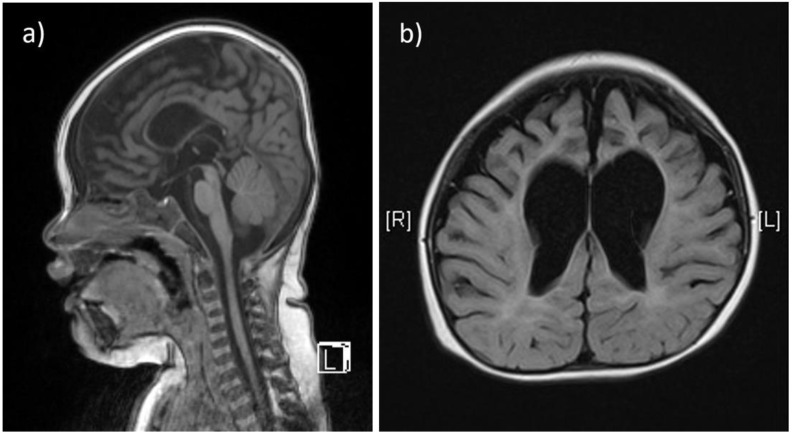
The T1-weighted, sagittal section of brain MRI from patient 1 shows thinning of the corpus callosum (**a**). The T2-weighted, axial section from the same patient shows diffuse cerebral atrophy involving both gray and white matters, and sulcal widening with prominent enlargement of the ventricular systems, especially at the frontal horns (**b**).

Two affected patients were born to healthy, non-consanguineous parents of Chinese descent. The first child (Patient 1) was a baby girl, born at 41 weeks of gestation by vaginal delivery, weighing 3300 g at birth. Prenatal exams were smooth, and the pregnancy was uneventful. She exhibited poor locomotive abilities a few months after birth, and had to rely on a nasogastric tube for feeding until 12 months. Staying floppy, she was unable to roll over, and exhibited progressive hypotonia and hyporeflexia in the extremities. Frequent generalized tonic-clonic seizure episodes (about every 1.5 h) were detected after 12 months, and she showed signs of further neurodegeneration since the onset of seizure episodes (stopped smiling and sucking, and lacked visual attention). She could make sounds without forming words. An X-ray revealed severe scoliosis and advanced bone age, more than 2 years older than chronological age. Brain MRI at 11 months showed thinning of the corpus callosum, diffuse cerebral atrophy involving both gray and white matters, and sulcal widening with prominent enlargement of the ventricular systems (Figure 1a,b). Other clinical exams showed thrombocytopenia and the presence of accessory spleen. Karyotyping analysis yielded normal results. She had recurrent respiratory infections and died of respiratory failure at 3 years of age.

**Figure 2:**
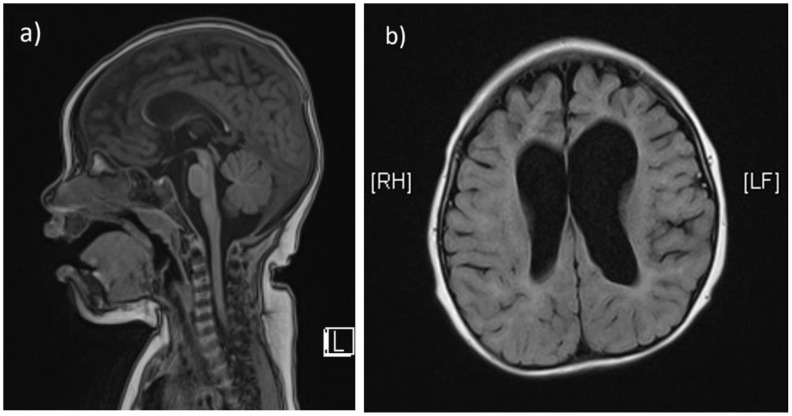
The T1-weighted, sagittal section of brain MRI from patient 2 also shows marked thinning of the corpus callosum (**a**). The T2-weighted, axial section shows prominent enlargement of cerebral cortical sulci and ventricles (**b**).

The second child (Patient 2) is a 2-year-old girl born at 39 + 6 gestational weeks via natural vaginal delivery, with a birthweight of 3800 g. Her prenatal and perinatal birth histories were unremarkable. She was initially brought to the hospital at around 12 months due to motor delay, as she was able to roll over, but unable to crawl, stand or stay sitting up unassisted. At 18 months, frequent seizures developed, ranging from focal to generalized tonic-clonic seizures, with irregular intervals (minutes to hours). Subsequently, a marked deterioration of muscle tone and neurologic regression was noted. She no longer smiled and became impassive to surroundings. She had no visual tracking and occasional nystagmus. She could produce vocal sounds without words. Laboratory test showed mild elevation of aspartate aminotransferase (37 IU/L) and CK (335 IU/L). EEG showed slow wave activities. A brain MRI performed at 12 months revealed hypoplasia of corpus callosum and prominent enlargement of cerebral cortical sulci and ventricles (Figure 2a,b). The patient and her family were referred for genetic survey after two consecutive probands suffering from similar debilitating neurodegenerative phenotypes at infancy. The clinical presentations of patient 1 and patient 2 are summarized in Table 1.

After obtaining written informed consent from the parents, genomic DNA was extracted from the peripheral blood and blood film of the patients and their parents. Whole-exome sequencing (WES) was performed on the affected sisters and their parents with Illumina NovaSeq 6000 sequencer using Agilent v6 Sureselect capture kit. Sequenced reads were aligned to human genome assembly GRCh37 (hg19) from the Genome Reference Consortium. HaplotypeCaller algorithm (GATK v. 3.4) was used for variant calling, followed by annotation with ANNOVAR. In-house scripted pipelines were applied for filtering and variant prioritization. We identified two suspect disease-causing *TBCD* variants: c.817+2T>C on intron 8, and c.1340C>T (p.Ala447Val) on exon 14 (NM_005993.4). Heterozygous *TBCD* c.817+2T>C mutation was present on patient 1, 2 and the unaffected father, while heterozygous c.1340C>T (p.Ala447Val) mutation was found on patient 1, 2 and the unaffected mother. The two variants were validated by PCR and Sanger sequencing.

## 3. Discussion

**Figure 3:**
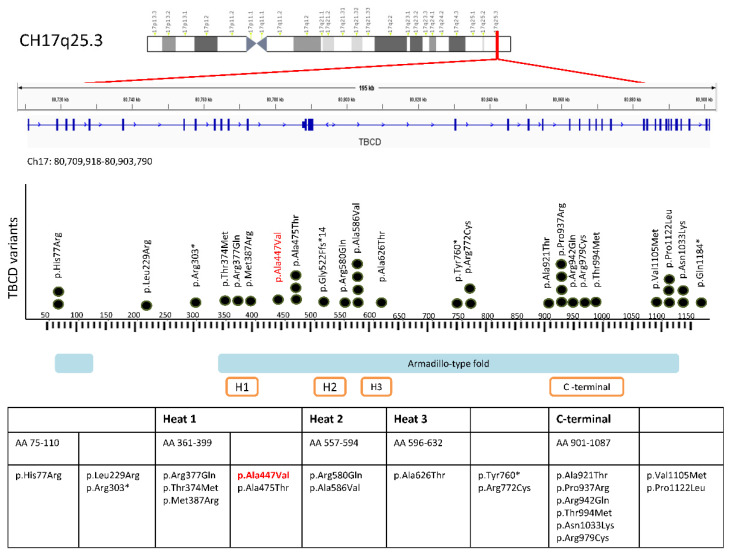
Schematic representation of *TBCD* gene located at chromosome 17q25.3, and its expressed *TBCD* protein product. The *TBCD* variants published in the previous literature associated with phenotypes of early-onset neurodegenerative encephalopathy are plotted in from N-terminal to C-terminal. Our reported variant is highlighted in red. Black dots represent the number of times a particular variant has been reported. Solid blue and orange-framed bars indicate the domains and repeated sequence motifs present in a *TBCD* protein. The insertion/deletion variant c.771+1_771+10del and splice site variants c.1564-12C>G, c.3192-2A>G, c.817+2T>C are not included. The star sign (*) denotes termination of the amino acid sequence, resulting in protein truncation.

Since 2016, there have been eight published reports of biallelic *TBCD* mutations associating with clinical features of neurodevelopmental impairment and early-onset neurodegenerative encephalopathy [2,3,4,5,6,7,8,9,10] (Figure 3).

Functional studies established the link between loss-of-function *TBCD* proteins and the diseased phenotype [3]. Wild-type *TBCD* proteins are integral to the axonal transport of mitochondria in neurons, and maintenance of proper growth and organization of mitotic spindles from the MTOC. Located in the centrosome, *TBCD* protein is one of five tubulin-specific chaperones (TBCA-E) that interact with other protein components of the microtubule network, namely β-tubulin, the regulatory GTPase ARL2, to form a functional protein complex. This quaternary structure is an intermediate scaffold, onto which α-tubulin is added, facilitated by other cofactors TBCE and TBCB [2,3]. When compared to the control, the fibroblast cell lines from the biallelic *TBCD* mutants exhibit a disorganized spindle structure, tangle-shaped mitotic microtubules, reduced aster formation, and delayed mitotic progression [2]. The disease-associated forms of *TBCD* also exhibited decreased thermal stability and lack of tubulin disassembly compared to the wild-type [2]. *TBCD* mutations were predicted to alter protein conformation and protein–protein interactions, and were more susceptible to degradation by the ubiquitin proteasome pathway [2,4].

A number of *TBCD* loss-of-function mutations have been linked to early-onset encephalopathy [2,3,4,6]. Biallelic mutations in *AGTPBP1*, a gene also involved in microtubule organization, exhibits similar features of childhood-onset neurodegeneration with cerebellar atrophy (CONDCA) in the absence of seizures [11]. The reported phenotypes of biallelic *TBCD* mutation include early-onset neurodevelopmental regression, epilepsy and hypotonia, which corresponded very closely to the clinical presentation observed in our affected cases. The compound heterozygous variants, c.817+2T>C on intron 8, and c.1340C>T (p.Ala447Val) on exon 14, have not been published in the previous literature (Table A1). The missense variant c.1340C>T has minor allele frequency (MAF) of 8.068E-6 according gnomAD population database (gnomAD v2.1.1), while the splicing-site variant c.817+2T>C was not listed on gnomAD, ClinVar or dbSNP. Segregation analysis of the parental DNA confirmed the unaffected mother to be a heterozygous carrier of c.1340C>T (p.Ala447Val), and the unaffected father to be a heterozygous carrier of c.817+2T>C, while the two affected children inherited both *TBCD* variants via autosomal recessive transmission. The amino acid substitution in missense variant c.1340C>T (p.Ala447Val) was predicted to be “damaging”, and “probably damaging” by multiple in silico tools such as SIFT, Polyphen-2 and MutationTaster. The missense variant c.817+2T>C was predicted to be pathogenic by computational tools BayesDel_addAF, DANN, EIGEN, FATHMM-MKL, MutationTaster, and scSNV-Splicing. SpliceAI v1.3 predicted a high probability of the variant being splice-altering, as the delta scores for donor gain (0.95) and donor loss (0.99) were both very high [12].

**Figure 4:**
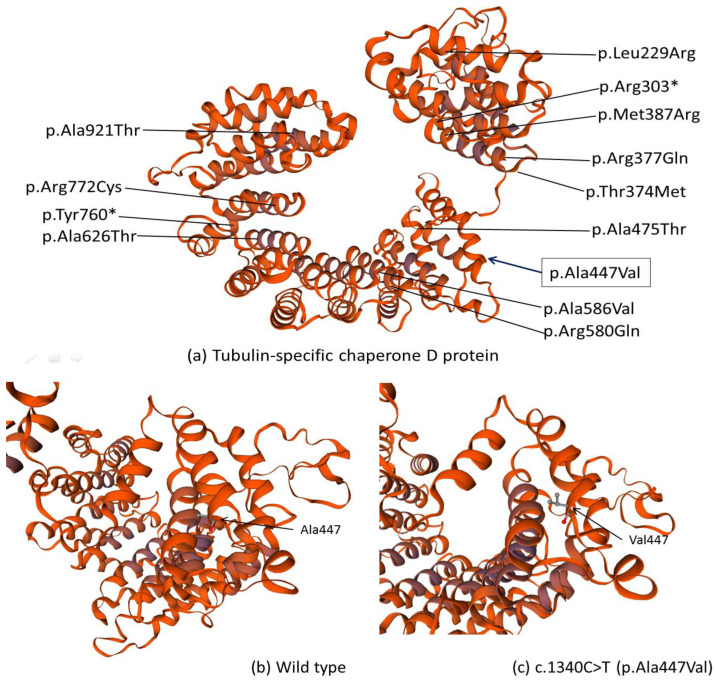
Homology-Modeled Structures of *TBCD* protein (UniProtKB AC: Q9BTW9) based on Swiss-model template (ID 1qgr.1.A, covering *TBCD* amino residues 194-931). (**a**) The secondary and tertiary structure prediction of *TBCD*; the locations of missense mutations previously published in past literatures are labeled. Variants that fall outside of this coverage are not shown. The location of our reported variant Ala447 is boxed. (**b**) shows the zoomed-in view of (**a**) at a different angle. The location of Ala447 in α-helix, the orientation and spacing of adjacent helices are labeled in (**b**) compared to its mutant form Val447 (**c**). The star sign (*) denotes termination of the amino acid sequence, resulting in a truncated protein.

The *TBCD* protein is mainly composed of armadillo/HEAT motifs, or multiple α-helices running in antiparallel directions linked by short loops [2] (Figure 4a). Structural impact of identified missense variant c.1340C>T (p.Ala447Val) was investigated with homology modeling using the SWISS-MODEL server (http://swissmodel.expasy.org, accessed on 8 July 2021.) [13,14]. By substituting Ala447, the best-helix-forming straight-chained residue, to an aliphatic β-branched amino acid Valine, the stability of the protein at the level of secondary and tertiary structures might be affected [15]. In the modeled *TBCD* tertiary structures, there appeared to be differences in the proximity and orientation to adjacent helices, probably due to steric effects [15] (Figure 4b,c) Previously, another compound heterozygous variant c.1757C>T (p.Ala586Val), resulting in Ala-to-Val substitution in a different α-helix of the *TBCD* protein, was reported [4,5], and this structural perturbation was also predicted to substantially affect the interactions between adjacent helices [4].

In conclusion, we identified two novel *TBCD* variants that were associated with the phenotype of early-onset progressive encephalopathy. Our findings strengthened the genotype–phenotype relationship between *TBCD*-mediated tubulinopathy and severe infantile neurodegenerative disorder. We also showed that *TBCD* protein function may be impacted, either directly or indirectly, by secondary and tertiary structure alteration following amino acid substitution.

## Figures and Tables

**Table 1:** Clinical presentation of patients with compound heterozygous *TBCD* mutations in this study.

	Patient 1	Patient 2
Gender, age	Female, 36 m (deceased, related to recurrent respiratory infection)	Female, 33 m
Age of seizure onset	12 m	18 m
Consanguinity	no	no
Perinatal history	unremarkable	unremarkable
Epilepsy	yes, GTS	yes, focal to GTS
Hypotonia	yes	yes
Swallowing	poor; NG feeding until 3 y	can eat solid food
Speech		
Skeletal involvement	scoliosis	no
Others	no	thrombocytopenia, accessory spleen

## Data Availability

The data presented in this study are available upon reasonable request from the corresponding author.

## Appendix A

**Table A1:** List of identified pathogenic *TBCD* variants associated with early-onset neurodegenerative encephalopathy in this study.

Physical Position (hg19)	Mutation Site	Amino Acid Change	Zygosity	MAF/TW	ClinVar	HGMD	SIFT/Polyphen-2	SpliceAI
17:80755680	c.817+2T>C	-	het	8.068E-6/3.3E-4	NA	NA	./.	DG 0.95, DL 0.99
17:80828121	c.1340C>T	p.Ala447Val	het	5.802E-05/NA	Likely Pathogenic	NA	D/P	-

Abbreviations: TBCD, tubulin Folding Cofactor D; hg19, human genome assembly GRCh37 (hg19) from Genome Reference Consortium; MAF, minor allele frequency based on dataset from gnomAD browser v2.1.1; TW, Taiwan biobank; HGMD, Human Gene Mutation Database; D, damaging; P, probably damaging; SpliceAIv1.3 annotations: DG, delta score for donor gain; DL, delta score for donor loss.

## References

[1] Lopez-Fanarraga M., Avila J., Guasch A., Coll M., Zabala J.C. (2001). Review: Postchaperonin tubulin folding cofactors and their role in microtubule dynamics. J. Struct. Biol..

[2] Flex E., Niceta M., Cecchetti S., Thiffault I., Au M.G., Capuano A., Piermarini E., Ivanova A.A., Francis J.W., Chillemi G. (2016). Biallelic Mutations in TBCD, Encoding the Tubulin Folding Cofactor D, Perturb Microtubule Dynamics and Cause Early-Onset Encephalopathy. Am. J. Hum. Genet..

[3] Miyake N., Fukai R., Ohba C., Chihara T., Miura M., Shimizu H., Kakita A., Imagawa E., Shiina M., Ogata K. (2016). Biallelic TBCD Mutations Cause Early-Onset Neurodegenerative Encephalopathy. Am. J. Hum. Genet..

[4] Pode-Shakked B., Barash H., Ziv L., Gripp K.W., Flex E., Barel O., Carvalho K.S., Scavina M., Chillemi G., Niceta M. (2017). Microcephaly, intractable seizures and developmental delay caused by biallelic variants in TBCD: Further delineation of a new chaperone-mediated tubulinopathy. Clin. Genet..

[5] Edvardson S., Tian G., Cullen H., Vanyai H., Ngo L., Bhat S., Aran A., Daana M., Da’amseh N., Abu-Libdeh B. (2016). Infantile neurodegenerative disorder associated with mutations in TBCD, an essential gene in the tubulin heterodimer assembly pathway. Hum. Mol. Genet..

[6] Zhang Y., Zhang L., Zhou S. (2020). Developmental Regression and Epilepsy of Infancy with Migrating Focal Seizures Caused by TBCD Mutation: A Case Report and Review of the Literature. Neuropediatrics.

[7] Ikeda T., Nakahara A., Nagano R., Utoyama M., Obara M., Moritake H., Uechi T., Mitsui J., Ishiura H., Yoshimura J. (2017). TBCD may be a causal gene in progressive neurodegenerative encephalopathy with atypical infantile spinal muscular atrophy. J. Hum. Genet..

[8] Tian D., Rizwan K., Liu Y., Kang L., Yang Y., Mao X., Shu L. (2019). Biallelic pathogenic variants in TBCD-related neurodevelopment disease with mild clinical features. Neurol. Sci..

[9] Stephen J., Nampoothiri S., Vinayan K.P., Yesodharan D., Remesh P., Gahl W.A., Malicdan M.C.V. (2018). Cortical atrophy and hypofibrinogenemia due to FGG and TBCD mutations in a single family: A case report. BMC Med. Genet..

[10] Grønborg S., Risom L., Ek J., Larsen K.B., Scheie D., Petkov Y., Larsen V.A., Dunø M., Joensen F., Østergaard E. (2018). A Faroese founder variant in TBCD causes early onset, progressive encephalopathy with a homogenous clinical course. Eur. J. Hum. Genet..

[11] Baltanás F.C., Berciano M.T., Santos E., Lafarga M. (2021). The childhood-onset neurodegeneration with cerebellar atrophy (Condca) disease caused by agtpbp1 gene mutations: The purkinje cell degeneration mouse as an animal model for the study of this human disease. Biomedicines.

[12] Jaganathan K., Kyriazopoulou Panagiotopoulou S., McRae J.F., Darbandi S.F., Knowles D., Li Y.I., Kosmicki J.A., Arbelaez J., Cui W., Schwartz G.B. (2019). Predicting Splicing from Primary Sequence with Deep Learning. Cell.

[13] Waterhouse A., Bertoni M., Bienert S., Studer G., Tauriello G., Gumienny R., Heer F.T., DeBeer T.A.P., Rempfer C., Bordoli L. (2018). SWISS-MODEL: Homology modelling of protein structures and complexes. Nucleic Acids Res..

[14] Studer G., Tauriello G., Bienert S., Biasini M., Johner N., Schwede T. (2021). ProMod3—A versatile homology modelling toolbox. PLoS Comput. Biol..

[15] Gregoret L.M., Sauer R.T. (1998). Tolerance of a protein helix to multiple alanine and valine substitutions. Fold. Des..

